# Reliability of Between-Session Motor Units Behavior: A Comparison of Analytical Approaches for Intervention Studies

**DOI:** 10.3390/sports14070305

**Published:** 2026-07-17

**Authors:** Daniel Marcos-Frutos, Kevin Méndez-Bouza, David Colomer-Poveda, Amador García-Ramos, Gonzalo Márquez

**Affiliations:** 1 Department of Physical Education and Sport, Faculty of Sport Sciences, University of Granada, 18071 Granada, Spain; amagr@ugr.es; 2 Department of Physical Education and Sport, Faculty of Sports Sciences and Physical Education, University of A Coruna, 15071 A Coruña, Spain; kevin.mendezb@udc.es (K.M.-B.); gonzalo.marquez@udc.es (G.M.); 3 Centre for Sport Studies, Rey Juan Carlos University, 28933 Madrid, Spain; david.colomerp@urjc.es; 4 Department of Sports Sciences and Physical Conditioning, Faculty of Education, Universidad Católica de la Santísima Concepción, Concepción 4090541, Chile

**Keywords:** motor control, neurophysiology, motor neurons, electromyography, strength training

## Abstract

Objective: To compare the between-session reliability of motor units (MUs) behavior derived from high-density surface electromyography (HD-sEMG) during isometric knee extension across three analytical approaches: all identified MUs averaged per participant, tracked MUs averaged per participant, and tracked MUs using individual MUs as the statistical unit. Methods: Seventeen resistance-trained volunteers completed one familiarization and two identical experimental sessions. HD-sEMG signals were assessed in vastus lateralis at 30%, 50%, and 70% of maximum voluntary torque (MVT). Plateau discharge rate, recruitment discharge rate, and recruitment threshold (expressed in %MVT and Nm) were calculated. Reliability was quantified with the Standard error of the measurement (SEM), Coefficient of variation (CV), and Intraclass correlation coefficient (ICC). Results: All identified MUs averaged per participant (CV = 11.7%, ICC = 0.73) yielded comparable reliability than tracked MUs averaged per participant (CV = 13.7%, ICC = 0.68). Using the individual MU as the statistical unit produced the lowest reliability (CV = 16.6%, ICC = 0.58). Conclusions: Averaging all identified MUs per participant achieved reliability comparable to tracking, without the associated sample loss or workload, supporting it as the preferable analytical approach for training interventions.

## 1. Introduction

High-density surface electromyography (HD-sEMG) techniques have been developed to non-invasively examine the behavior of individual motor units (MUs) [1]. This approach is particularly valuable for tracking adaptations in muscle function and neuromuscular control following interventions such as strength training [2,3]. Additionally, the relatively large number of MUs identified with HD-sEMG allows for potentially robust and representative data across multiple sessions [4]. However, several factors may influence HD-sEMG-derived MUs behavior, including skin preparation, subcutaneous tissue thickness, MU spatial distribution, muscle cross-sectional area, electrode grid placement and orientation, signal conditioning, and manual editing of MUs spike trains, among others [5,6,7]. Therefore, to successfully detect changes in MUs behavior after training interventions, it is essential to confirm that MUs decomposition methods yield consistent results across different measurement sessions (i.e., reliability).

To address these reliability concerns, previous studies have assessed the consistency of HD-sEMG-derived MUs behavior. Martínez-Valdés et al. [4] reported good to excellent within- and between-session reliability of MU behavior in the vastus lateralis and medialis. Later, Martínez-Valdes et al. [8] showed that longitudinal MU tracking improves between-session reliability compared to analyzing all identified or only untracked MUs in vastus lateralis and medialis. Goodlich et al. [9] tracked MUs within-session (using blind source separation filters) and between-session (using waveform cross-correlation) in the tibialis anterior, reporting comparable reliability across methods and between pharmacological and placebo conditions. Notably, Goodlich et al. [9] reported lower reliability at higher contraction intensities. Lecce et al. [10] reported high Intraclass correlation coefficient (ICC) values across pre- to post-intervention for tracked MUs in the biceps brachii, in both the trained and untrained limbs following 4 weeks of cross-education. Finally, Hug et al. [11] showed that both expert and novice operators achieved similar results when manually editing MU spike trains, supporting inter-operator reliability.

While these studies provide valuable insights into MUs spike train editing and MUs behavior reliability, a further consideration that has received insufficient attention is the choice of statistical unit. Some studies have analyzed MUs as the statistical unit [12,13], which risks biasing results towards participants with a higher number of decomposed MUs. However, other studies have averaged MUs behavior per participant [2,14,15], which provides a summary of neuromuscular control at a given intensity but necessarily obscures the behavior of specific MUs subtypes (e.g., low- vs. high-threshold). These two analytical approaches may yield different reliability estimates, and it remains unclear which is more appropriate for between-session designs aimed at detecting training-induced adaptations in MUs behavior. Directly comparing both approaches is therefore necessary to inform the methodological choices of future exercise training studies using HD-sEMG.

An additional approach used in training intervention studies is to longitudinally track MUs across sessions [3,10,16,17,18]. Longitudinal tracking aims to detect the same MU across sessions [8]. Therefore, it could be considered as a more robust approach to detect changes in MUs behavior, since the same population of MUs is being compared across time points rather than two possibly different populations, as occurs when analyzing the whole pool [3,8]. On the other hand, one of the main limitations of longitudinal tracking is the substantial sample loss associated with the tracking procedure in intervention studies, which can range from 50 to 85% sample loss when tracking across two time points [3,8,10,16,18,19] or 88% across three time points [17], which can reduce both statistical power and the physiological characterization of training-induced changes in MUs behavior. The last is particularly relevant when the final number of tracked MUs per participant is very low (e.g., a single tracked MU is used to represent an entire participant’s adaptation). Whether applying longitudinal tracking or comparing the whole identified pool, and whether averaging MU behavior per participant or using individual MUs as the statistical unit, could differentially affect between-session reliability. Directly comparing these analytical combinations could therefore help identify the most appropriate methodological approach for exercise training studies using HD-sEMG.

To contribute to these gaps, this study aimed to compare the between-session reliability of HD-sEMG-derived MUs behavior during isometric knee extension (KE) across three analytical approaches: using all identified MUs and averaging values per participant, tracking MUs and averaging values per participant, and tracking MUs and using individual MUs as the statistical unit. Notably, using all identified MUs and using individual MUs as the statistical unit was not feasible for reliability estimates since they require paired observations. We hypothesized that between-session reliability would be higher for tracked MUs, given that the same MU population is compared across sessions, and for analyses using individual MUs as the statistical unit, given the larger sample size compared to averaging per participant.

## 2. Materials and Methods

### 2.1. Experimental Protocol

This study employed a crossover design with one familiarization session and two experimental sessions, each separated by 3–5 days. Participants were instructed to avoid demanding physical activity for 48 h and caffeine consumption for 24 h before each session. The first session was used to familiarize participants with the study procedures by the experimental sessions. In the experimental sessions, participants performed several isometric trapezoidal contractions at different intensities of KE. The order of the intensities was randomized and counterbalanced with the Research Randomizer online software (www.randomizer.org). To test reliability, both experimental sessions were identical. All testing sessions were conducted in the university’s research laboratory at consistent times (±1 h) for each participant.

### 2.2. Participants

Seventeen resistance-trained volunteers (one female) participated in the study (25.1 ± 4.0 years, 176.9 ± 8.2 cm, 75.7 ± 10.3 kg, all but one right-leg dominant). MUs identification was not possible in one male participant. Inclusion criteria require participants to be 18–40 years old and to have a minimum of one year of resistance training experience. Exclusion criteria included any underlying health issues and a history of traumatic lower body and/or spinal injury. The study adhered to the Declaration of Helsinki and was approved by the Research Ethics Committee of A Coruña-Ferrol (2022/309). All participants provided written informed consent after being briefed on the study’s objectives and experimental procedures.

### 2.3. Experimental Sessions

#### 2.3.1. Neuromuscular Assessment

Participants were seated in an isokinetic machine (HUMAC NORM Isokinetic Dynamometer, CSMI, Stoughton, MA, USA) to measure right leg KE torque. Both knees remained flexed at 90°, hips flexed at ~100° (i.e., slightly over the perpendicular) to ensure no knee extensors activity at the beginning. The torso was restrained with belts to avoid any displacement. The warm-up consisted of several 3 s KE isometric contractions: 2 sets of 3 repetitions at 30%, 2 sets of 3 repetitions at 50%, 2 repetitions at 70% and 1 repetition at 90% of self-perceived Maximal voluntary torque (MVT). After warm-up, participants performed 2 maximal voluntary contractions (MVC) of 5 s with a 3 min rest in-between to determine MVT. They were verbally encouraged to “push as hard as possible”.

**Figure 1:**
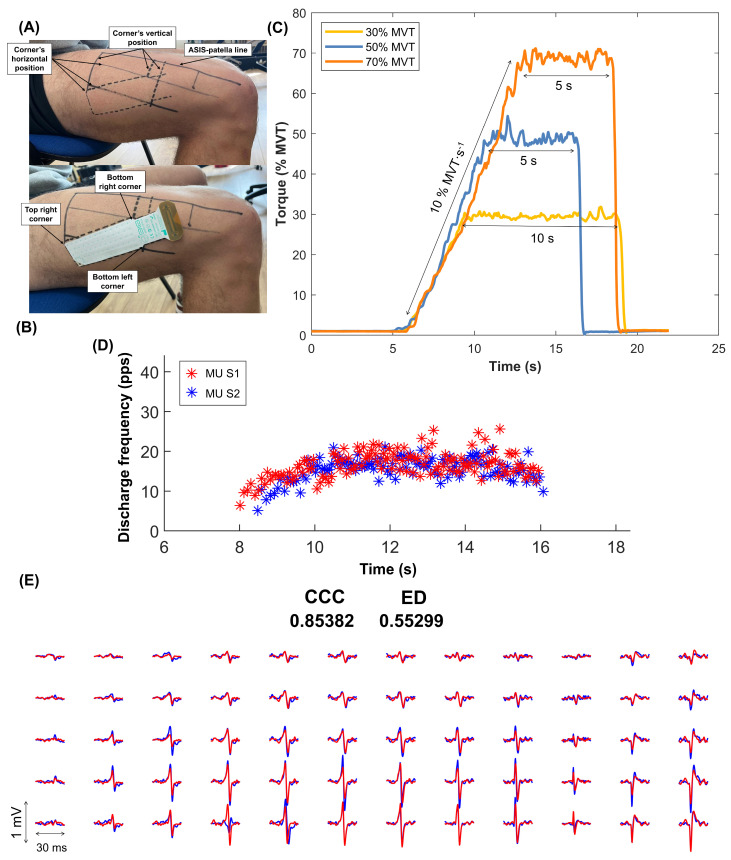
(**A**,**B**) Illustration of grid anatomical landmarks used for grid placement from first to second experimental sessions. First, a line from the anterior superior iliac spine to the superolateral border of the patella was drawn (ASIS-patella line). Second, horizontal lines were marked at the vertical positions (“heights”) of the top-right, bottom-right, and bottom-left grid corners. Third, vertical lines were marked at the horizontal distance of each corner from the ASIS-patella line. These horizontal and vertical coordinates for the three corners were then used to reposition the grid in the second session. (**C**) Torque-time curves of isometric knee extension trapezoidal contractions at 30%, 50%, and 70% of the maximum voluntary torque (MVT) performed by one participant. (**D**) Instantaneous discharge frequencies of two tracked motor units (MUs) at 30% MVT across sessions 1 (S1) and 2 (S2). (**E**) MU action potentials from the same MUs shown in panel (**D**), including cross-correlation coefficient (CCC) and Euclidean distance (ED) values. Red color lines show the first trial, and blue color the second trial.

A non-recorded for analysis trapezoidal contraction at 30% MVT was performed 3 min after MVC to reinforce technical execution. After a 90 s rest, participants performed 6 isometric trapezoidal contractions, all registered for analysis. Trapezoidal contractions were performed at 30%, 50%, and 70% MVT (2 repetitions per intensity) with 90 s, 2 min and 3 min rests, respectively. The rate of torque development during the ascending ramp was fixed at 10% MVT·s^−1^ for all intensities. The plateau phase lasted 10 s at 30% MVT and 5 s at both 50% and 70% MVT. There was no descending ramp (Figure 1C).

#### 2.3.2. HD-sEMG and Torque Recording

EMG activity of the right vastus lateralis was recorded using a high-density surface adhesive grid with 64 electrodes (13 rows × 5 columns, 1 mm electrode diameter, 8 mm inter-electrode distance; GR08MM1305, OT Bioelettronica, Torino, Italy). As anatomical landmarks, the grid was placed at: (a) the proximal two-thirds of the line from the anterior superior iliac spine (ASIS) and superolateral border of the patella and (b) at 6.5 cm horizontally from that line, as confirmed in a pilot study to correspond approximately to the innervation zone. Furthermore, the B-mode option of an ultrasound device (Versana Active, GE Healthcare, Chicago, IL, USA) with a 38 mm linear-array probe (6–13 MHz) was used to determine muscle fascicle orientation (i.e., grid orientation). Participants were seated with knees at 90°, as this would be the same position during the assessments. A generous amount of transmission gel (GIMA, Gessate, Italy) was applied to exert minimal pressure. The exact location was corrected from the familiarization session to the first experimental session after checking familiarization results to ensure that the grid was placed centrally over the innervation zone and oriented with muscle fascicle orientation (determined by B-mode ultrasound). Before grid placement, skin was shaved, abraded with paste, and cleaned with alcohol. To optimize the skin-to-electrode contact, disposable bi-adhesive foam layers with cavities filled with conductive paste (SpesMedica, Battipaglia, Italy) were used to attach the grid to the skin. To determine between-session reliability, grid was carefully placed in the same position in the two experimental sessions. Several anatomical landmarks, including the horizontal and vertical position of the top right, bottom right and bottom left grid corners with respect to the ASIS-patella line, were measured twice to ensure the same position in both sessions (Figure 1A,B).

All signals were analog-to-digital converted (Quattrocento, OT Bioelettronica), amplified by a factor of 150, sampled at 2048 Hz, and band-pass filtered (10–500 Hz, first order, −3 dB). HD-sEMG signals were acquired in monopolar mode. A wet strap was positioned below the tested knee, at the level of the patellar tendon and popliteal fossa, for high-density grid reference. Three additional wet straps were placed at the same location on the contralateral knee and on both ankles over the malleoli, serving as main ground electrodes. The grid and all the ground electrodes were connected to the same EMG amplifier. The torque was synchronized with the HD-sEMG signal through the auxiliary input of the EMG amplifier. The signals were recorded with the software OT BioLab+ 1.5.9.0 (OT Bioelettronica) and stored on a PC for offline processing and analyses.

### 2.4. Data Analysis

#### 2.4.1. Signals Processing and HD-sEMG Decomposition

The torque signal was low-pass filtered (10 Hz, fourth-order Butterworth) and normalized to the MVT recorded on the same day. The HD-sEMG signal was visually inspected using a custom-made MATLAB script (R2020a, The MathWorks, Inc., Natick, MA, USA). Channels exhibiting excessive noise (resting root mean square > 30 µV) were removed (<2.5% channels removed). Following inspection, the HD-sEMG signal was band-pass filtered (10–900 Hz, fourth-order Butterworth) and notch-filtered at 50 Hz to attenuate power line interference. MUs spike trains were then extracted using a blind source separation algorithm, which enables the automatic identification of multiple individual MUs [20]. Decomposition accuracy was estimated with the silhouette measure, which quantifies the contrast between the spike train peaks relative height and the baseline noise [20]. The signal was decomposed throughout the whole duration of the submaximal contractions, and the discharge times of the identified MUs were converted into binary spike trains [8,20].

All discharge times were inspected and manually edited by the same operator using a custom-made MATLAB script. Missing spikes producing non-physiological discharge rates (i.e., interspike intervals > 250 ms) were manually and iteratively excluded, and the spike train was recalculated. In addition, in cases where the algorithm incorrectly assigned multiple spikes to a single discharge, erroneous spikes were removed, and the final spike trains were re-estimated [5]. The plateau discharge rate was defined as the average discharge rate during the plateau phase of the contraction [21]. The recruitment discharge rate was defined as the average discharge rate of the first six discharges [22]. The recruitment threshold was defined as both the normalized (%MVT) and absolute (Nm) torque value at the time of the first detected MU discharge [21].

#### 2.4.2. Motor Unit Longitudinal Tracking

MUs were tracked across experimental sessions using a developed method [8]. This method identifies MUs with MU action potential (MUAP) shapes that remain maximally similar across sessions (Figure 1D,E). The MUAP waveforms were extracted from the decomposed signals using the discharge times provided by the decomposition algorithm. MUAP similarity was quantified using the waveform cross-correlation coefficient (CCC), which measures temporal and shape alignment between MUAPs. Additionally, a Euclidean distance (ED)-based metric was used, calculated point-by-point across the entire MUAP waveform and channels, where values close to 1 indicate maximal similarity and values close to 0 indicate minimal similarity. Only MUs with a CCC greater than 0.7 [2,3,18] and a normalized ED greater than 0.5 were considered as tracked. In cases where a given MU met these criteria with multiple other MUs, the candidate MU with the highest combined CCC and ED similarity was retained as the tracked MU.

### 2.5. Statistical Analysis

From the two trials recorded per intensity, only the one with the greater number of decomposed MUs after manual editing was retained for longitudinal tracking and statistical analyses. Three analytical conditions were compared for between-session reliability: (1) all identified MUs averaged per participant as the statistical unit (N = number of participants), (2) tracked MUs averaged per participant as the statistical unit (N = number of participants), and (3) tracked MUs using individual MUs as the statistical unit (N = total number of tracked MUs across participants).

Data normality was assessed using the Shapiro–Wilk’s test. Paired samples *t*-test was used to explore between-session differences in MVT. Statistical significance was set at *p* < 0.05. Between-session reliability for each analytical approach was evaluated using the Standard error of the measurement (SEM), within-subjects Coefficient of variation (CV) and ICC (model 3.1), with the CV calculated based on the SEM. CV values were categorized as low (≥10%), acceptable (5–10%), and high (<5%) reliability [23]. ICC values were classified as poor (<0.50), moderate (0.50–0.75), good (0.75–0.90), and excellent (>0.90) reliability [24]. Reliability analyses were conducted using a customized Microsoft Excel spreadsheet (Microsoft 365, v.2412, Redmond, WA, USA) [25], while all other statistical analyses were performed using SPSS software version 23.0 (SPSS Inc., Chicago, IL, USA).

## 3. Results

**Figure 2:**
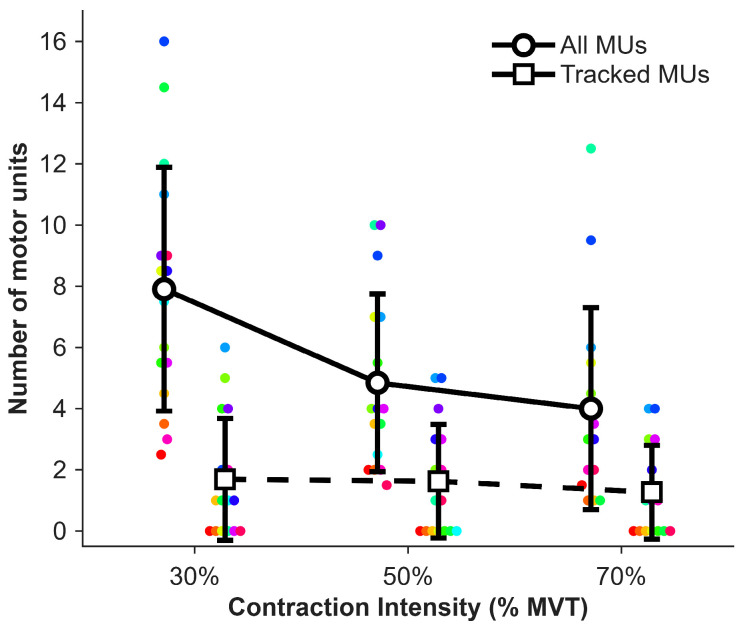
Mean ± standard deviation of number of motor units (MUs) identified per participant. For each intensity, the left column (circles) shows the number of all identified MUs after manual editing, averaged between Session 1 and Session 2, and the right column (squares) shows the number of MUs successfully tracked between sessions. Colored dots represent individual participants. MVT: Maximal voluntary torque.

No significant between-session differences were observed in MVT (278.81 ± 88.89 Nm vs. 290.29 ± 92.10 Nm, *p* = 0.104). The average number of identified MUs per participant decreased from 7.9 to 4.0 as contraction intensity increased (Figure 2). The average number of tracked MUs per participant was more stable across intensities (N = 1.3–1.6), although the final tracked sample was substantially lower at all intensities than the total number of identified MUs (66–78% sample loss). The CCC and ED of tracked MUs are reported in Table 1.

### 3.1. All Identified Motor Units, Averaged per Participant

Table 2 presents the between-session reliability of all identified MUs, averaged per participant. SEM ranged from 0.8 to 1.9 pps for the discharge rate variables and 1.9–6.7%MVT (5.5–21.0 Nm) for recruitment threshold. Five CVs indicated acceptable reliability (CV = 7.1–8.9%), while seven represented low reliability (CV = 10.9–18.8%). ICC values were poor in two cases (ICC = 0.43–0.47), moderate in four (ICC = 0.57–0.73), good in five (ICC = 0.83–0.87), and excellent in one (ICC = 0.93).

### 3.2. Tracked Motor Units, Averaged per Participant

Table 3 presents the between-session reliability of tracked MUs, averaged per participant. SEM ranged from 1.0 to 2.0 pps for the discharge rate variables and 4.4–19.8 (%MVT or Nm) for recruitment threshold. Only one CV indicated acceptable reliability (CV = 7.3%), with the remaining eleven values representing low reliability (CV = 10.2–22.9%). ICC values were poor in four cases (ICC = 0.15–0.46), good in seven (ICC = 0.78–0.88), and excellent in one (ICC = 0.94).

### 3.3. Tracked Motor Units, Individual Motor Units as the Statistical Unit

Table 4 presents the between-session reliability of individually tracked MUs (MU as the statistical unit). SEM ranged from 1.1 to 2.0 pps for the discharge rate variables and 4.8–30.4 (%MVT or Nm) for recruitment threshold. Two CVs indicated acceptable reliability (CV = 6.9–9.4%), while the remaining ten represented low reliability (CV = 11.4–25.0%). ICC values were poor in four cases (ICC = 0.20–0.46), moderate in five (ICC = 0.51–0.57), and good in three (ICC = 0.72–0.80).

## 4. Discussion

Overall, HD-sEMG-derived MUs behavior showed moderate between-session reliability, although this varied considerably depending on the analytical approach and the variable. Averaging MUs behavior per participant, whether using all identified MUs or tracked MUs, yielded comparable reliability, with neither approach showing a consistent advantage over the other. In contrast, using individual MUs as the statistical unit yielded the lowest reliability overall, despite the larger sample size, suggesting that the increase in N did not compensate for the greater dispersion introduced by treating each MU as an independent observation. Reliability also depended on the variable assessed, being consistently higher for plateau discharge rate than for recruitment-phase variables, and higher for recruitment threshold when expressed in absolute (Nm) rather than relative (%MVT) terms. Based on these findings, averaging MUs behavior per participant using all identified MUs, without longitudinal tracking, appears to be the most suitable approach for between-session designs aimed at detecting training-induced adaptations, as it achieved comparable reliability to the tracked-MU approach while avoiding the additional sample loss and the time-consuming tracking procedure.

### 4.1. Comparison of Analytical Approaches for Between-Session Reliability

Averaging MU behavior per participant produced comparable between-session reliability whether all identified MUs (Table 2; CV = 11.7% [7.1–18.8%], ICC = 0.73 [0.43–0.93]) or only tracked MUs were used (Table 3; CV = 13.7% [6.6–22.9%], ICC = 0.68 [0.15–0.94]). This result was contrary to our hypothesis that tracking would improve reliability by comparing the same MU population across sessions. Using the individual MU as the statistical unit yielded the lowest reliability (Table 4; CV = 16.6% [6.9–25.0%], ICC = 0.58 [0.20–0.80]) despite the larger sample size (N = 19–27).

Our between-session reliability was generally lower than that reported by previous studies [4,8,10], and several methodological factors likely contribute to this divergence. A first candidate is grid repositioning. Lecce et al. [10] assessed the biceps brachii, which is an easier muscle for grid placement, and traced anatomical landmarks onto individual acetate sheets. Martinez-Valdes et al. [8] assessed vastus lateralis and vastus medialis and marked grid position on the skin with a surgical pen. On the other hand, we relied on anatomical landmark measurements for vastus lateralis. Between-session MUAP reproducibility is highly sensitive to grid placement [5], and the lower number of MUs we tracked compared with Martinez-Valdes et al. [8] (22–34% versus 25–58%) might be consistent with this explanation. This suggests that marking grid placement with a surgical pen or acetate tracing should be preferred for between-session studies aiming to track MUs.

However, this explanation is not sufficient on its own. Goodlich et al. [9] also marked grid position with a surgical pen yet reported higher CVs (10.1–32.2%) than us (6.6–25.0%) at comparable testing intensities, indicating that a surgical pen does not guarantee better reliability in every case. Additionally, our CCC for tracked MUs (81.5–83.3%) was only slightly lower than that reported by Martinez-Valdes et al. [8] (81.0–86.9%), despite our presumably less precise grid repositioning. If anatomical landmarks are indeed less reproducible than surgical pen marking, a high CCC obtained under less precise positioning raises the possibility of false positives. That is, MUs being accepted as matched because they have a high CCC despite not being the same MU.

Comparability across studies is further limited by inconsistent reporting. Neither Lecce et al. [10] nor Goodlich et al. [9] reported the achieved CCC of their final tracked MUs sample, which prevents a direct check of whether their tracking accuracy was comparable to ours. Similarly, neither Goodlich et al. [9] nor Martinez-Valdes et al. [8] explicitly stated whether the participant or the individual MU was used as the statistical unit for their reliability analyses, which, as shown in the present study, can affect the resulting CVs and ICCs.

Finally, these reliability estimates should also be interpreted with appropriate caution. With 10 to 19 participants contributing data across all reliability studies [8,9,10], including the present work, reliability metrics can be unstable with such small samples [26,27]. The divergent conclusions across studies regarding whether tracking improves reliability appear to reflect differences in grid placement method, muscle, variable type, statistical unit, reporting practices, and small samples analyzed, which underscores the need for standardized methodologies and reporting practices in this field with larger sample sizes. Additionally, the differences between the analytical approaches observed in this study should therefore be read as broadly comparable rather than as definitive rankings, given this imprecision to obtain tighter estimates. However, this reinforces, rather than weakens, the practical message that tracking offered no clear advantage over the all-MU approach due to the substantial sample loss that longitudinal tracking yielded in the present study but also in prior work (50–88% loss) [2,3,8,9,10,16,17,18,19].

### 4.2. Reliability Across Variables

Reliability depended strongly on the variable assessed. Plateau discharge rate was the most reliable variable across all three approaches; whereas, recruitment discharge rate and recruitment threshold were consistently less stable. This pattern is methodologically coherent. Plateau discharge rate is averaged over a multi-second window of steady force [21] and is therefore more robust. In contrast, the recruitment-phase variables depend only on one or a few numbers of discharges [21], which makes them more sensitive to random variability.

The recruitment threshold illustrated a dissociation between CVs and ICCs. The absolute threshold (Nm) showed higher ICCs (0.51–0.94) than the relative threshold (%MVT; ICC = 0.15–0.82) despite comparable CVs (8.9–25.0% vs. 8.5–23.5%). This is caused by the distinction of the two forms of reliability [28]. The ICC is a relative index that reflects the ratio of between-subject variance to total variance, so it increases when participants differ markedly from one another. Normalizing the threshold to MVT removes between-subject differences in strength, which compresses the between-subject variance and lowers the ICC without reducing the measurement error itself. The high ICC of the absolute threshold therefore reflects large between-subject differences in strength rather than superior within-subject consistency. In contrast, the CV, an absolute index, is more informative for judging whether a real within-subject change can be detected and suggested similar measurement error across recruitment thresholds.

### 4.3. Limitations and Future Directions

Several limitations should be acknowledged. First, the sample was modest, which produced wide confidence intervals, so the present findings should be generalized cautiously. Second, grid repositioning relied on anatomical landmarks rather than permanent skin marking or acetate tracing, which probably contributed to the lower between-session reliability of the longitudinal tracking. Third, only one muscle (vastus lateralis) and a single task (isometric knee extension) were examined, and MUs behavior and measurement reliability may differ between muscles and tasks. Fourth, no a priori power calculation was performed, and the present sample size was instead guided by comparable HD-sEMG reliability studies. Fifth, the sample was almost exclusively male (one female). Therefore, the findings should be generalized mainly to young resistance-trained men. Future reliability studies should employ larger samples and adopt standardized methodology and reporting such as specifying the statistical unit and the CCC values after tracking, ensuring consistent grid repositioning, and extending the assessment to multiple muscles and tasks.

## 5. Conclusions

Between-session reliability of vastus lateralis MUs behavior during isometric knee extension was moderate and varied with both the analytical approach and the variable assessed. Averaging MUs behavior per participant with all identified MUs achieved reliability comparable to longitudinal tracking, while avoiding the associated sample loss and tracking workload. Therefore, all identified MUs averaged per participant is recommended as the default approach for training interventions, particularly when only a small number of MUs are tracked. These conclusions rest on a modest sample with wide confidence intervals and warrant confirmation in larger studies.

## Figures and Tables

**Table 1:** Cross-correlation coefficients and Euclidean distances of tracked motor units.

Intensity (% MVT)	CCC (%)	ED (%)
30	83.28 ± 3.97	56.21 ± 4.33
50	82.84 ± 4.63	57.42 ± 5.08
70	81.49 ± 4.29	56.94 ± 4.42

Data are presented as mean ± standard deviation. MVT: Maximal voluntary torque; CCC: cross-correlation coefficient; ED: Euclidean distance.

**Table 2:** Between-session reliability of all identified motor units, averaged per participant.

Variable	Intensity	N	Session 1	Session 2	SEM	CV	ICC
Plateau DR (pps)	30	16	10.5 ± 1.4	10.4 ± 1.9	0.9 (0.7, 1.4)	8.5 (6.3, 13.2)	0.73 (0.39, 0.90)
	50	15	12.7 ± 1.9	13.3 ± 2.7	0.9 (0.7, 1.5)	7.1 (5.2, 11.2)	0.87 (0.65, 0.95)
	70	14	17.0 ± 3.7	17.2 ± 5.0	1.9 (1.4, 3.1)	**11.3 (8.2, 18.2)**	0.83 (0.56, 0.94)
Recruitment DR (pps)	30	16	9.2 ± 1.4	9.3 ± 2.0	0.8 (0.6, 1.2)	8.3 (6.1, 12.8)	0.83 (0.57, 0.94)
	50	15	10.7 ± 1.4	10.4 ± 1.6	1.2 (0.8, 1.8)	**10.9 (8.0, 17.2)**	**0.43 (−0.08, 0.77)**
	70	14	11.4 ± 3.0	11.4 ± 2.0	1.5 (1.1, 2.4)	**13.0 (9.4, 20.9)**	0.69 (0.28, 0.89)
Recruitment threshold (% MVT)	30	16	22.0 ± 2.9	22.0 ± 2.6	1.9 (1.4, 2.9)	8.5 (6.3, 13.1)	0.57 (0.11, 0.82)
	50	15	33.5 ± 5.3	31.7 ± 5.9	4.2 (3.1, 6.6)	**12.8 (9.4, 20.2)**	**0.47 (−0.03, 0.79)**
	70	14	41.2 ± 12.2	39.1 ± 12.2	6.7 (4.9, 10.8)	**16.7 (12.1, 26.9)**	0.73 (0.35, 0.90)
Recruitment threshold (Nm)	30	16	60.5 ± 19.6	62.6 ± 19.1	5.5 (4.0, 8.4)	8.9 (6.5, 13.7)	0.93 (0.81, 0.98)
	50	15	96.7 ± 36.2	92.3 ± 33.9	15.2 (11.1, 24.0)	**16.1 (11.8, 25.4)**	0.83 (0.58, 0.94)
	70	14	114.6 ± 58.9	109.0 ± 48.4	21.0 (15.2, 33.9)	**18.8 (13.6, 30.3)**	0.87 (0.64, 0.96)

Data are presented as mean ± standard deviation or mean (95% confidence interval). Bold values indicate CV ≥ 10% or ICC < 0.50. N: number of participants with motor units decomposed in both sessions; SEM: Standard error of the measurement; CV: Coefficient of variation; ICC: Intraclass correlation coefficient; DR: discharge rate; MVT: Maximal voluntary torque.

**Table 3:** Between-session reliability of tracked motor units, averaged per participant.

Variable	Intensity	N	Session 1	Session 2	SEM	CV	ICC
Plateau DR (pps)	30	10	11.1 ± 2.2	10.7 ± 2.6	1.2 (0.8, 2.2)	**11.0 (7.6, 20.1)**	0.80 (0.38, 0.95)
	50	9	13.3 ± 2.0	13.4 ± 2.7	1.0 (0.7, 1.9)	7.3 (5.0, 14.0)	0.87 (0.54, 0.97)
	70	8	16.2 ± 2.3	15.4 ± 2.7	1.0 (0.7, 2.1)	6.6 (4.4, 13.5)	0.88 (0.51, 0.97)
Recruitment DR (pps)	30	10	9.7 ± 2.0	10.0 ± 3.1	2.0 (1.4, 3.7)	**20.4 (14.0, 37.2)**	**0.46 (−0.20, 0.83)**
	50	9	10.9 ± 1.2	10.4 ± 1.4	1.1 (0.7, 2.1)	**10.2 (6.9, 19.6)**	**0.34 (−0.37, 0.80)**
	70	8	12.3 ± 3.5	11.8 ± 2.2	1.5 (1.0, 3.1)	**12.5 (8.3, 25.4)**	0.80 (0.30, 0.96)
Recruitment threshold (% MVT)	30	10	19.9 ± 6.1	21.8 ± 3.8	4.8 (3.3, 8.7)	**22.9 (15.8, 41.8)**	**0.15 (−0.50, 0.69)**
	50	9	33.7 ± 4.8	34.1 ± 6.6	4.4 (2.9, 8.4)	**12.9 (8.7, 24.6)**	**0.49 (−0.20, 0.86)**
	70	8	45.6 ± 13.0	46.4 ± 11.5	6.0 (4.0, 12.2)	**13.1 (8.6, 26.6)**	0.82 (0.35, 0.96)
Recruitment threshold (Nm)	30	10	66.0 ± 23.7	72.4 ± 22.4	12.0 (8.3, 22.0)	**17.4 (12.0, 31.8)**	0.78 (0.33, 0.94)
	50	9	88.2 ± 30.1	88.3 ± 29.6	13.3 (9.0, 25.6)	**15.1 (10.2, 29.0)**	0.85 (0.47, 0.96)
	70	8	130.7 ± 70.3	128.2 ± 63.8	19.8 (13.1, 40.3)	**15.3 (10.1, 31.2)**	0.94 (0.75, 0.99)

Data are presented as mean ± standard deviation or mean (95% confidence interval). Bold values indicate CV ≥ 10% or ICC < 0.50. N: number of participants with motor units decomposed in both sessions; SEM: Standard error of the measurement; CV: Coefficient of variation; ICC: Intraclass correlation coefficient; DR: discharge rate; MVT: Maximal voluntary torque.

**Table 4:** Between-session reliability of individually tracked motor units (N = motor units).

Variable	Intensity	N	Session 1	Session 2	SEM	CV	ICC
Plateau DR (pps)	30	27	11.2 ± 2.3	10.6 ± 2.3	1.2 (1.0, 1.7)	**11.4 (9.0, 15.6)**	0.72 (0.48, 0.86)
	50	26	13.5 ± 2.2	13.3 ± 2.4	1.3 (1.0, 1.7)	9.4 (7.4, 13.0)	0.72 (0.46, 0.86)
	70	19	16.7 ± 2.3	16.2 ± 2.5	1.1 (0.9, 1.7)	6.9 (5.2, 10.2)	0.80 (0.55, 0.92)
Recruitment DR (pps)	30	27	9.7 ± 2.1	9.8 ± 2.6	1.6 (1.2, 2.2)	**16.1 (12.7, 22.1)**	0.57 (0.25, 0.78)
	50	26	10.9 ± 2.2	10.1 ± 1.5	1.4 (1.1, 1.9)	**13.2 (10.4, 18.3)**	**0.46 (0.10, 0.72)**
	70	19	11.8 ± 3.0	11.7 ± 2.0	2.0 (1.5, 3.0)	**17.0 (12.9, 25.2)**	**0.41 (−0.04, 0.72)**
Recruitment threshold (% MVT)	30	27	19.9 ± 5.4	21.4 ± 5.2	4.8 (3.8, 6.5)	**23.1 (18.2, 31.7)**	**0.20 (−0.19, 0.53)**
	50	26	33.2 ± 6.2	32.2 ± 8.4	5.6 (4.4, 7.7)	**17.0 (13.3, 23.4)**	**0.45 (0.08, 0.71)**
	70	19	42.5 ± 13.1	41.9 ± 14.2	9.3 (7.0, 13.8)	**22.1 (16.7, 32.7)**	0.56 (0.15, 0.80)
Recruitment threshold (Nm)	30	27	68.8 ± 19.1	74.5 ± 21.4	14.4 (11.4, 19.8)	**20.2 (15.9, 27.6)**	0.51 (0.17, 0.74)
	50	26	94.9 ± 32.3	90.4 ± 34.5	16.1 (12.6, 22.2)	**17.3 (13.6, 23.9)**	0.78 (0.57, 0.90)
	70	19	126.1 ± 61.6	117.1 ± 59.0	30.4 (23.0, 45.0)	**25.0 (18.9, 37.0)**	0.77 (0.49, 0.90)

Data are presented as mean ± standard deviation or mean (95% confidence interval). Bold values indicate CV ≥ 10% or ICC < 0.50. N: Number of participants with motor units decomposed in both sessions; SEM: Standard error of the measurement; CV: Coefficient of variation; ICC: Intraclass correlation coefficient; DR: discharge rate; MVT: Maximal voluntary torque.

## Data Availability

Source data are available from the corresponding author upon request.

## Abbreviations

The following abbreviations are used in this manuscript:

ASIS	Anterior superior iliac spine
CCC	Cross correlation coefficient
CV	Coefficient of variation
ED	Euclidean distance
EMG	Electromyography
HD-sEMG	High-density surface electromyography
ICC	Intraclass correlation coefficient
KE	Knee extension
MU	Motor unit
MUAP	Motor unit action potential
MVC	Maximum voluntary contraction
MVT	Maximum voluntary torque
SEM	Standard error of the measurement

## References

[1] Drost G., Stegeman D.F., van Engelen B.G.M., Zwarts M.J. (2006). Clinical Applications of High-Density Surface EMG: A Systematic Review. J. Electromyogr. Kinesiol..

[2] Casolo A., Farina D., Falla D., Bazzucchi I., Felici F., Del Vecchio A. (2020). Strength Training Increases Conduction Velocity of High-Threshold Motor Units. Med. Sci. Sports Exerc..

[3] Del Vecchio A., Casolo A., Negro F., Scorcelletti M., Bazzucchi I., Enoka R., Felici F., Farina D. (2019). The Increase in Muscle Force after 4 Weeks of Strength Training Is Mediated by Adaptations in Motor Unit Recruitment and Rate Coding. J. Physiol..

[4] Martinez-Valdes E., Laine C.M., Falla D., Mayer F., Farina D. (2016). High-Density Surface Electromyography Provides Reliable Estimates of Motor Unit Behavior. Clin. Neurophysiol..

[5] Del Vecchio A., Holobar A., Falla D., Felici F., Enoka R.M., Farina D. (2020). Tutorial: Analysis of Motor Unit Discharge Characteristics from High-Density Surface EMG Signals. J. Electromyogr. Kinesiol..

[6] Merletti R., Cerone G.L. (2020). Tutorial. Surface EMG Detection, Conditioning and Pre-Processing: Best Practices. J. Electromyogr. Kinesiol..

[7] de Oliveira D.S., Casolo A., Balshaw T.G., Maeo S., Lanza M.B., Martin N.R.W., Maffulli N., Kinfe T.M., Eskofier B.M., Folland J.P. (2022). Neural Decoding from Surface High-Density EMG Signals: Influence of Anatomy and Synchronization on the Number of Identified Motor Units. J. Neural Eng..

[8] Martinez-Valdes E., Negro F., Laine C.M., Falla D., Mayer F., Farina D. (2017). Tracking Motor Units Longitudinally across Experimental Sessions with High-Density Surface Electromyography. J. Physiol..

[9] Goodlich B.I., Del Vecchio A., Kavanagh J.J. (2023). Motor Unit Tracking Using Blind Source Separation Filters and Waveform Cross-Correlations: Reliability under Physiological and Pharmacological Conditions. J. Appl. Physiol..

[10] Lecce E., Amoruso P., Del Vecchio A., Casolo A., Felici F., Farina D., Bazzucchi I. (2025). Neural Determinants of the Increase in Muscle Strength and Force Steadiness of the Untrained Limb Following a 4 Week Unilateral Training. J. Physiol..

[11] Hug F., Avrillon S., Del Vecchio A., Casolo A., Ibanez J., Nuccio S., Rossato J., Holobar A., Farina D. (2021). Analysis of Motor Unit Spike Trains Estimated from High-Density Surface Electromyography Is Highly Reliable across Operators. J. Electromyogr. Kinesiol..

[12] Lecce E., Casolo A., Nuccio S., Felici F., Bazzucchi I. (2026). Analysis of Motor Units with High-Density Surface Electromyography: Methodological Considerations and Physiological Significance. Eur. J. Appl. Physiol..

[13] Chen C., Liu X., Qiu F. (2025). Characterization of Motor Unit Activities during Isometric Elbow Flexion with Different Speeds. J. Neuroeng. Rehabil..

[14] Casolo A., Del Vecchio A., Balshaw T.G., Maeo S., Lanza M.B., Felici F., Folland J.P., Farina D. (2021). Behavior of Motor Units during Submaximal Isometric Contractions in Chronically Strength-Trained Individuals. J. Appl. Physiol..

[15] Contreras-Hernandez I., Arvanitidis M., Falla D., Negro F., Martinez-Valdes E. (2024). Achilles Tendon Morpho-Mechanical Parameters Are Related to Triceps Surae Motor Unit Firing Properties. J. Neurophysiol..

[16] Casolo A., Del Vecchio S., Goodlich B.I., Schrader B., Nuccio S., Lecce E., Bazzucchi I., Angius L., Felici F., Schrader J. (2026). Ageing Does Not Impair Motor Neuron Adaptations: Comparable Motor Unit Responses to Strength Training in Young and Older Adults. J. Physiol..

[17] Lecce E., Conti A., Del Vecchio A., Felici F., Scotto di Palumbo A., Sacchetti M., Bazzucchi I. (2025). Cross-Education: Motor Unit Adaptations Mediate the Strength Increase in Non-Trained Muscles Following 8 Weeks of Unilateral Resistance Training. Front. Physiol..

[18] Andrews M.H., Martinez-Valdes E., Lichtwark G.A., Pincheira P.A. (2026). Biceps Femoris Long-Head Motor Unit Discharge Rates and Recruitment Thresholds Vary across 9 Weeks of Eccentric Training. J. Sport Health Sci..

[19] Valli G., Sarto F., Casolo A., Del Vecchio A., Franchi M.V., Narici M.V., De Vito G. (2024). Lower Limb Suspension Induces Threshold-Specific Alterations of Motor Units Properties That Are Reversed by Active Recovery. J. Sport Health Sci..

[20] Negro F., Muceli S., Castronovo A.M., Holobar A., Farina D. (2016). Multi-Channel Intramuscular and Surface EMG Decomposition by Convolutive Blind Source Separation. J. Neural Eng..

[21] Valli G., Ritsche P., Casolo A., Negro F., De Vito G. (2024). Tutorial: Analysis of Central and Peripheral Motor Unit Properties from Decomposed High-Density Surface EMG Signals with Openhdemg. J. Electromyogr. Kinesiol..

[22] Farina D., Holobar A., Gazzoni M., Zazula D., Merletti R., Enoka R.M. (2009). Adjustments Differ Among Low-Threshold Motor Units During Intermittent, Isometric Contractions. J. Neurophysiol..

[23] Miras-Moreno S., García-Ramos A., Sašek M., Cvjetičanin O., Šarabon N., Kavčič I., Smajla D. (2024). Individual Acceleration-Speed Profile Variables: Comparison and Reliability Between Linear and Curvilinear Sprints. J. Strength Cond. Res..

[24] Koo T.K., Li M.Y. (2016). A Guideline of Selecting and Reporting Intraclass Correlation Coefficients for Reliability Research. J. Chiropr. Med..

[25] Hopkins W. (2015). Spreadsheets for Analysis of Validity and Reliability. Sportscience.

[26] Schönbrodt F.D., Perugini M. (2013). At What Sample Size Do Correlations Stabilize?. J. Res. Pers..

[27] Warneke K., Keiner M., Wallot S., Siegel S.D., Günther C., Wirth K., Puschkasch-Möck S. (2025). The Impact of Sample Size on Reliability Metrics Stability in Isokinetic Strength Assessments: Does Size Matter?. Meas. Phys. Educ. Exerc. Sci..

[28] Hopkins W.G. (2000). Measures of Reliability in Sports Medicine and Science. Sports Med..

